# The complete chloroplast genome sequence of *Photinia × fraseri,* a medicinal plant and phylogenetic analysis

**DOI:** 10.1080/23802359.2021.1972874

**Published:** 2021-09-15

**Authors:** Huixing Li

**Affiliations:** a Henan Key Laboratory of Industrial Microbial Resources and Fermentation Technology, Nanyang Institute of Technology, Nanyang, Henan, China; b School of Biological and Chemical Engineering, Nanyang Institute of Technology, Nanyang, Henan, China

**Keywords:** *Photinia × fraseri*, chloroplast genome, phylogenetic analysis

## Abstract

*Photinia × fraseri* is a common ornamental arbor in the genus *Photinia* (family Rosaceae), which complete chloroplast (cp) genome was sequenced, assembled and annotated. The chloroplast genome of *P. fraseri* was 160,184 bp in length, including a large single copy (LSC) region of 88,121, a small single copy (SSC) region of 19,295 bp, and two inverted repeat (IR) regions of 26,384 bp. The GC contents of LSC, SSC, IR and whole genome are 36.5%, 34.1%, 30.3%, and 42.7%, respectively. There are 131 genes annotated, including 84 protein-coding genes, 37 tRNA genes, and 8 rRNA genes. The phylogenetic analysis revealed that *P. fraseri* was most related to *Photinia serratifolia* as a sister group with 100% bootstrap support. The complete chloroplast genome sequences of *P. fraseri* will provide valuable genomic information to further illuminate phylogenetic classification of *Photinia* genus.

The genus *Photinia* belongs to Maleae (Rosaceae) and comprises approximately 60 species (Lu and Spongberg 2003). They are widespread landscape tree species resistant to pruning and air pollution (Mori et al. 2018), and many were cultivated for gardening (Zhao et al. 2020). *Photinia × fraseri* is an evergreen plant species, which grows in thickets at altitudes of 600–1000 m and mainly distributes in southern China and Southeast Asia (Lu and Spongberg 2003). Like many other species of *Photinia*, *P. fraseri* has luxuriant foliage around the trunk, purple and tender leaves in early spring, and little white flowers in early summer, and bear red fruits in autumn (Mattei et al. 2018). *Photinia* species exhibited similar morphological features and the species boundaries have been unclear. With the continuous discovery of new *Photinia* species (Li et al. 2015), a reliable classification of *Photinia* is in urgent need. For a better understanding of the relationships of *P. fraseri* and other *Photinia* species, it is necessary to reconstruct a phylogenetic tree based on high-throughput sequencing approaches. In this present study, we reported and characterized the complete chloroplast (cp) genome of *P. fraseri* based on Illumina pair-end sequencing and compared it with other genus cp genome sequences. The result would supply valuable information for the evolution process and conservation genetics of *P. fraseri*.

The sample of *P. fraseri* was collected from Nanchong, Sichuan province, China (106°08′E; 30°79′N). A specimen was deposited at the herbarium of Henan Key Laboratory of Industrial Microbial Resources and Fermentation Technology, Nanyang Institute of Technology (http://www.nymc.edu.cn/, Li Huixing, 3592264762@qq.com) under the voucher number HYSN001. The DNA sample was properly stored at Key Laboratory of Nanyang Institute of Technology, Nanyang, China. Total genomic DNA was extracted using the DNA Secure Plant Kit (Tiangen Biotech, Beijing, China) following the manufacturer’s protocol. Library preparation and genomic sequencing on the Illumina Hiseq 2500 platform were conducted by Benagen (Benagen Inc., Wuhan, China). The raw sequence data has been deposited into NCBI SRA with project accession of SRR14793491. The raw data was filtered using Trimmomatic Version 0.32 with default settings (Bolger et al. 2014). The filtered output was a 5.4 Gb raw data of 150 bp paired-end reads. The obtained paired-end reads were assembled using SPAdes v.3.9.0 (Nurk et al. 2017). The assembled sequence was annotated in MPI-MP CHLOROBOX (https://chlorobox.mpimp-golm.mpg.de/geseq.html) via GeSeq with the reference cp genome of *P. serratifolia* (NC045331), and then corrected using Geneious Prime v2020.2. Finally, the complete chloroplast genome of *P. fraseri* was submitted to GenBank (Accession No. MZ128520).

The chloroplast genome of *P. fraseri* was 160,184 bp in length, including a large single copy (LSC) region of 88,121, a small single copy (SSC) region of 19,295 bp, and two inverted repeat (IR) regions of 26,384 bp. The GC contents of LSC, SSC, IR and whole genome are 36.5%, 34.1%, 30.3%, and 42.7%, respectively. There are 131 genes annotated, including 84 protein-coding genes, 37 tRNA genes, and 8 rRNA genes.

To further investigate its taxonomic status, a maximum-likelihood (ML) tree was constructed based on complete chloroplast genome sequences using MEGA 7.0 (Kumar et al. 2016) with 1000 bootstrap replicates. The program operating parameters were set as follows: a Tamura 3-parameter (T92) nucleotide substitution model with 1000 bootstrap repetitions, accompanied by Gamma distributed with Invariant site (G + I) rates, and partial deletion of gaps/missing data. We used the complete chloroplast genomes sequence of P. fraseri and 16 other related species to construct phylogenetic tree. Information of the 16 related species is shown in Figure 1. The 17 chloroplast genome sequences were aligned with MAFFT (Katoh and Standley 2013), and then the maximum-likelihood (ML) tree was constructed. The phylogenetic analysis revealed that *P. fraseri* was most related to *P. serratifolia* as a sister group with 100% bootstrap support. The complete chloroplast genome sequences of *P. fraseri* will provide valuable genomic information to further illuminate phylogenetic classification of Photinia genus.

**Figure 1.**
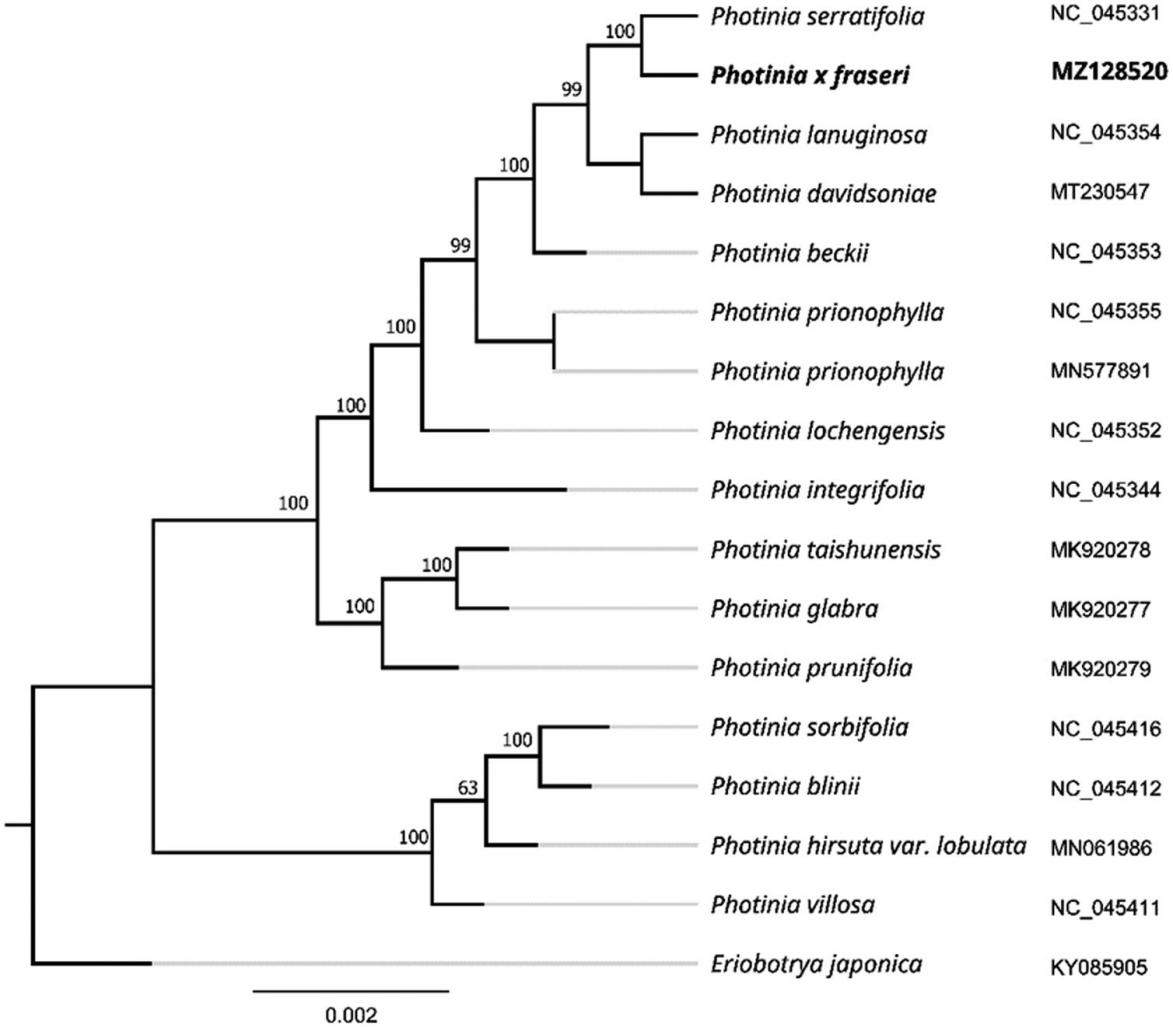
Maximum likelihood phylogenetic tree of *Photinia × fraseri* and other related species based on the complete chloroplast genome sequence.

## Data Availability

The genome sequence data that support the findings of this study are openly available in GenBank of NCBI at (https://www.ncbi.nlm.nih.gov/) under the accession No. MZ128520. The associated BioProject, SRA, and Bio-Sample numbers are PRJNA737038, SRR14793491, and SAMN19678360 respectively.
